# Supplementary material for: Correlates of Hepatitis B infection in pregnant women attending antenatal clinics in Wa Municipality, Ghana
Source: PLOS Glob Public Health. 2024 Sep 10;4(9):e0002447. doi: 10.1371/journal.pgph.0002447 (PMC11386425; doi:10.1371/journal.pgph.0002447)
Supplement: Attachment — Submitted filename: Response to Reviewers_PGPH-D-23-01433.docx [file pgph.0002447.s001.docx]

Response to Reviewers:

Reviewer #1

Overall, there are some typos that need to be checked and corrected in the entire text. For instance, "highly transmissible" in the third line of the abstract should be followed by a comma before the word "especially".

Thank you for this observation. We have inserted the punctuation as recommended. Please refer to line 20 in the abstract of the version with tracked changes.

The statement "A prospective systematic random cross-sectional study was conducted" in the methods section of the abstract appears to be vague. Is it a prospective systematic random study in terms of the design or the sampling approach?
It will be lucidly comprehended if it is rephrased as follows: A prospective cross-sectional study was conducted among 183 systematically and randomly selected pregnant women. Alternatively, it could be rephrased in a more canonical way by saying that "a prospective cross-sectional study employing systematic random sampling was conducted among 183 pregnant women attending..."

The reviewer is right about this oversight, and we appreciate it. We have considered the recommendation and revised the statement as suggested. Please see the methods section (lines 25-26) in the abstract of the version with tracked changes.

In the results section of the abstract, did you mean to say "being in a polygamous marital affair or relationship compared to monogamy?". If yes, kindly correct the current statement as it states "being in a polygamous marital compared to monogamy".

Yes, we meant to say being in a polygamous marital relationship compared to monogamy. We have revised the statement according to the suggestion. See lines 45-50 in the version with tracked changes.

Is it the virus that is transmitted vertically (i.e., materno-foetally) or the surface antigen? Kindly recheck and probably correct the last statement in the conclusion section of the abstract.

Thank you for this observation. It is indeed the virus that is transmitted vertically. However, since the presence of the surface antigen is a classic indication of an active and transmissible hepatitis B virus, and as there likely cannot be surface antigen without the virus itself, it is common to see the usage of the surface antigen to proxy the transmission of the virus itself. Nevertheless, we have revised the statement as recommended. Please see the conclusion of the abstract with tracked changes (line 54).

If the statement "...especially in sub-Saharan Africa (5%) including Ghana, even though it is twice as much (10%) in Asia" is referring to HBV infection burden (i.e., prevalence), then you should attribute the figures to the regional prevalence of HBV across SSA and Asia, respectively, instead of inserting the figures without indicating what they describe.

The reviewer has observed correctly – we meant the burden of the diseases in the two continental regions. The preceding part of the statement clearly mentioned that “...the disease has become more prevalent, life-threatening, and a tremendous public health challenge…”, which clarifies that we refer to the burden of the disease. We also categorically indicated the proportions corresponding to SSA and Asia. Thus, we believe there is enough clarity that the figures sufficiently describe/refer to the prevalence of the disease across the two regions.

There seems to be a conflicting sampling technique reported to have been used for participants' selection. At first, it was stated that a systematic random sampling approach was employed, and then again, consecutive sampling. If a systematic random sampling technique was used, was there a sampling frame? And what was the fixed sampling interval that followed the random start, if any (which wasn't mentioned, of course)? However, if it is consecutive sampling, then the statement on systematic random sampling should be corrected.

The reviewer is correct about the potential conflict in sampling technique, and we appreciate the attentive nature of the observation. We have now revised the statement to ensure sufficient clarity of the technique. Please see lines 25-26 in the abstract of the manuscript with tracked changes.

Regarding the sample size estimation, kindly include or reference the value of the proportion of the population attribute (i.e., p in the Cochran formula) that yielded a sample size of 183, if the confidence interval is at 95% and the margin of error is 5%. Did you in any way factor in the non-response proportion in the final sample size employed?

We thank the reviewer for this significant observation. Yes, we factored a 5% nonresponse rate into achieve the final sample size. We have now revised the methods section in the manuscript to reflect these important details for clarity. Please see lines 133-137 of the manuscript with tracked changes.

There is no statement or empirical evidence about the sensitivity and specificity of the employed Wondfo Rapid One-Step HBsAg test kit. This is very important for the validity of this study and for future studies that would want to leverage the same methods.

We agree with and thank the reviewer for this important observation. We have revised the script (lines 158-160 in tracked changes) to include the sensitivity and specificity ratings of the Wondfo One Step rapid diagnostic test kit.

Since you do not report having a reference scale for the assessment of HBV-specific knowledge, it will be better if you do not use the terms "assessment" and "knowledge," as written in the data collection section of the methodology: "assessment of their knowledge of mother-to-child transmission and prevention of HBV." Hence, the word "awareness" should be uniformly used since it is obvious that you sought to capture their awareness of some characteristic features of the infection, such as transmission and prevention.
Furthermore, the awareness variable was only reported descriptively using frequency and proportions. Yet, in the methods section of the abstract, it was claimed that the variable was assessed using an awareness summary score. Where is the summary or the weighted aggregate score computed for the variable and the reference scale for the assessment? Therefore, the appropriate word or words should be used in place of "assessment".

The reviewer is right about this observation, and we have revised the statements (lines 150-151 in tracked changes) accordingly. As indicated in the results (Table 2, and lines 246-247 in tracked changes) of this paper, we used the word “awareness” instead of assessment. So, we inconsistently interchanged the use of “knowledge” and “awareness”.

Kindly rephrase the first statement under the "Data Analysis" section so that charts and tables will not appear as forms of descriptive statistics. They are both for data presentation.
-Which type of Chi-square test did you employ?
-What was the basis for the selection of just two variables for the multi-variable logistic model?

Thank you for this observation. We appreciate the reviewer’s effort in this regard, but we maintain that tables and charts are both used for descriptive statistics data and are appropriate as presented in this study. Nevertheless, we have revised the statement under Data analysis. Regarding the type of Chi-square test, we used the test of independence/association in this study’s analysis, as already indicated in the last part of the last sentence under data analysis.

Basis for two variables in the multiple regression model. We acknowledge the significance of the reviewer’s observation. We are aware that using only two variables in the multiple logistic regression model sounds counterintuitive. However, we added only the two variables for two reasons:

1. To avoid overfitting: because our sample size is small, adding all the variables to the model could result in overfitting, where the model becomes too closely fitted to the specific sample and may not generalize well to the population. By limiting the number of variables, we aimed to reduce the risk of overfitting and ensure the model's applicability to a broader context.
2. Simplicity and Clarity: we focused on two variables to simplify the interpretation of the model - making it easier to understand the direct relationship between these variables and the outcome. We feel this approach is particularly useful because the primary objective aims to highlight their relationship with the outcome variable, without the potential confounding effects of the other variables.

How did you ensure confidentiality regarding the participants' HBV test results and other data captured? Kindly include this in the ethical consideration section.

This is an important piece of information, and we thank the reviewer for drawing our attention to it. We have revised the ethical consideration section (lines 200-204) to include this information.

I think it is appropriate to express the last category of the respondents age as ≥35 (i.e., 35 years and above), instead of (35+) −44 in table 1. Because if the oldest respondents were aged 44 years and you wanted it to reflect in the age categorization, then you could have expressed the last age group as 35–44, similar to other categories.
-In the heading of Table 4, do you mean multiple/multivariable logistic regression instead of "multi-logistic regression"? Please check.

Thank you for this observation. You are right and we have revised the age group across all tables (where applicable) to reflect the recommendation. Regarding Table 4, we have also revised the heading to reflect the appropriate terminology (multivariable logistic regression).

Check and correct the typos in this statement in the discussion section: "However, as recorded elsewhere, there was no significant association between respondents’ age group and their HB HBsAg-positivity status in this study, unlike reported elsewhere [21]".

This is an important observation, and we thank the reviewer for the high level of vigilance. We have revised the statement to reflect what we originally intended. Please lines 294-295 in tracked changes in the discussion section.

"More than half of the pregnant women in the study area were HBsAg-positive". Really? Is 20.2% (i.e., HBsAg-positive results) more than half? Kindly correct the statement.

Again, this was a significant oversight we missed. We appreciate the keenness of the reviewer in pointing out these errors. We have revised the statement to reflect the reality of findings in this study. Please refer to the opening statement in the conclusion section.

Any study limitations?

We thank the reviewer for drawing our attention to the absence of limitations in our study. We acknowledge the significance of indicating study limitations viz-a-viz the interpretation and applicability of the findings. As such, we have added a section on the limitations of our study. Please refer to lines 341-354 in the tracked changes manuscript.

**Reviewer #2:** TITLE: The title would be better rendered as "Factors associated with Hepatitis B viral infection among pregnant women attending antenatal care in Wa Municipality, Ghana".

We agree with and appreciate the reviewer’s suggestion and have revised the title of the study. Even though it does not exactly as the reviewer framed it, we believe it accurately reflects the nature of our study and conveys the message appropriately.

A. OBSERVATIONS: These are presented below;

Abstract: The objective of the study in line 4 of the abstract would greatly benefit from a revision and it is suggested that it should read thus; "This study sought to determine factors associated with the prevalence of Hepatitis B infection among pregnant women in Wa Municipality of Ghana."
The methods section of the abstract should read thus; "A cross-sectional survey study design was conducted among 183 consenting pregnant women in nine antenatal care facilities in Wa Municipality. A structured validated questionnaire was used to collect information about socio-demographic and obstetric characteristics, awareness of HBV transmission and its prevention scores. Blood samples (3.0 mls) were collected from each participant to test for HBV serum markers using a Wondfo One Step HBV rapid immunochromatographic assay (Catalog number W003) for the Hepatitis B surface antigen (HBsAg) profile. Data was analyzed using Stata/SE 15. Descriptive statistics were used for the socio-demographic, prevalence, and awareness variables. Chi-square and Fisher´s Exact Test were performed to determine the association between the variables. The result section appear flawed considering that values of the 95% confidence intervals reported In lines 3-6 are inappropriately stated without the sample statistic and actually reflect not directly association but prediction computed from binary logistic regression analysis which is not mentioned as one of the statistical tools used. In actual fact, relationships are best expressed with regression analysis and the corresponding predictive values of the explanatory variables computed with binary logistic regression. These analysis are inadequate.

Thank you very much for the detailed observations and suggestions. We have revised the various sections of the abstract as suggested. We wish to clarify that test of association was performed using Chi-square. Basically, we tested to know whether there was a significant association between the covariates and Hepatitis B infection status without necessarily quantifying the strength or direction of the association. We acknowledge and appreciate the reviewer’s concern on the result in this regard, but we, respectfully, maintain that the analysis done, and the results presented are sufficient for this paper based on the limitation of the study sample as indicated. Please refer to Table 3 for the detail results.

INTRODUCTION: The major revisions are needed for the introduction for observed typographical issues and lack of adequate conceptualization of modifiable risk factors of the at-risk population and their personal-level disposition that puts them at risk of an infection such as perception of the risks and consequences of an infection which includes transmitting the virus to their infants, attitudinal disposition towards preventing becoming infected and that prevention is far better than cure. These are salient factors to be considered in such a study. Throughout the introduction, the study did not address likely sources of associated modifiable and non-modifiable risk factors for HBV infection. In addressing these, how do you intend to establish scientific basis for these risk factors and demonstrate association as indicated by the title of the study? The contents of the study are basically what constitute risk factors for infection. No conceptual and theoretical framework or structured questions raised for the study to answer. Since these were not considered before planning the study protocol, it would mean going back to the drawing board and fresh data collection.

We greatly appreciate the reviewer’s observations regarding the Introduction. We acknowledge that we could have written it much better to also highlight certain risk factors associated with viral hepatitis B transmission in the selected cohort. Consequently, we have made relevant adjustments to the introduction of this study to cater for the highlighted concern. Respectfully, however, we do not think that the concern requires a re-visitation of the drawing board for fresh data collection. The aspect of risk factors in this study is a secondary outcome measure, and inasmuch as it deserves equal significance of attention, adjusting the literature to reflect its inclusion in the study does not need fresh data collection. Please refer to introduction of the manuscript in tracked changes to see highlights of the changes effected.

Methodology:
There is no evidence to suggest that the study design is a prospective study design because data collection does not resemble such hence it is recommended to stay with "facility-based cross-sectional survey design…".

Thank you for this prompt. We agree with the suggestion and have revised the statement to reflect accordingly.

Kindly be informed that Cochrane does not have a sample size computational formula, but this study is merely citing "sample size formula used in a study conducted by Cochrane in 1977" Kindly state this to reflect as expressed above which is the preferred syntax. Variables in the study appears not to be informed by any structured theoretical framework to confer validity to the instrument for data collection. The observed inclusion of knowledge in the study as a factor. Data analysis is insufficient because only a few variables have been considered. Data analysis is inadequate and lack rigor such study deserves.

The reviewer is correct about the Cochrane 1977 regarding sample size computation. We have adjusted the statement to reflect the suggested expression. Please see lines 134-139 in tracked changes.

Additionally, the reviewer is right about the number of variables we considered for the multi-logistic regression. We wish to highlight that we considered and included only those variables which showed statistical significance (i.e., age with positive HBeAg, and marital type (monogamy vs. polygamy) with positive HBsAg, HBeAg and HBcAg (see Table 3)) were necessary for inclusion in the analytical model. Variables which showed insignificant statistical association with out our outcome were deemed irrelevant for the model. For this reason, we respectfully maintain that this was sufficient and for this study.

Discussions:
The discussion demonstrates clearly that the study was mainly laboratory study and was unable to uncover adequately the associated factors involved with observed prevalence of HBV infection among participants in the study. While knowledge was identified as a factor in the infection dynamics related to personal-level disposition of the at-risk population, this is not sufficient. The lack of adequately exploring fully important factors can be seen from the inability of the study to conceptualize the likely dynamics of infectivity within the epidemiological principles of the natural history of HBV infectivity, the public health principles of prevention and control and the theoretical framework of the modifiable risk factors of the population of interest. Therefore, a lot of factors have not been considered in this study. This makes the study weak and unable to provide proof of concept in regard to the objective of the study.

With the conclusion stating that "More than half of the pregnant women in the study area were HBsAg-positive…" and that "Facility and community-based antenatal health education should also focus on increasing maternal knowledge of the mother-to-child transmission route of HBV" would not solve the problem because awareness alone is not sufficient to trigger the necessary caution and infection-prevention decision necessary to establish behaviour that translates to prevention of infection and transmission of the virus. The conclusion raises more questions than answers. It is not enough to test the population to determine the level of infection within the community but what may be responsible for this situation.

We appreciate the reviewer’s observation and recommendation regarding the discussion. We do acknowledge the fact that some factors known to be significant within the epidemiological principle of infectivity have not been fully explored in this study, and we highlight this in the limitation section. However, it is also important to note that every study considers the factors that are inherently considerable within the significant limits of the study, aside from the existing general knowledge of their relevance. Thus, it is impossible for every study to include every factor known to be significant in the infectivity dynamic of epidemiology.

The study’s conclusion of “more than half of the pregnant women in the study area were HBsAg-positive…" was a typo and has now been rectified. Additionally, we agree that the recommendation that “Facility and community-based antenatal health education should also focus on increasing maternal knowledge of the mother-to-child transmission route of HBV” alone would not solve the problem. For this reason, we first emphasized that “...the findings of this study highlight the need for routine screening and vaccination of pregnant women, particularly those at high risk, to prevent the transmission of hepatitis B viral infection to their offspring.” Please refer to conclusion section in the tracked changes manuscript.
