# Supplementary material for: Correlates of Hepatitis B infection in pregnant women attending antenatal clinics in Wa Municipality, Ghana
Source: PLOS Glob Public Health. 2024 Sep 10;4(9):e0002447. doi: 10.1371/journal.pgph.0002447 (PMC11386425; doi:10.1371/journal.pgph.0002447)
Supplement: Attachment — Submitted filename: Response to Reviewers_PGPH-D-23-01433R1.docx [file pgph.0002447.s002.docx]

Dear professor Nnodimele Onuigbo Atulomah (PhD),

We would like to thank you for overseeing this review process and the reviewers for their constructive comments on our manuscript entitled " *Factors associated with Hepatitis B viral infection among pregnant women attending antenatal care in Wa Municipality, Ghana* (Submission ID PGPH-D-23-01433)."

We have addressed each of the concerns of the reviewers. Please see below for details. We would be happy to make any further revisions as required. Thank you for your consideration.

**Simon Aabalekuu**

**(**on behalf of co-authors)

**Responses to editor/reviewers Comments**

***Comments***

This is an important study that needs to be published to provide the understanding the authors of this study desire to share. The authors' concerns are well justified. However, there are certain minor but very important syntax issues to be resolved and straightened out to make the argument in the manuscript credible. Some were pointed out in the first round of the manuscript review but were not attended to. Please attend to these observed issues.

1) The title has been revised as recommended.

**Response:** We thank the editor and reviewers for this.

2) Abstract: The recommendation made by one of the reviewers to modify the methods section to read: "A cross-sectional survey study design was conducted among 183 consenting pregnant women in nine antenatal care facilities in Wa Municipality. A structured validated questionnaire was used to collect information about socio-demographic and obstetric characteristics, awareness of HBV transmission and its prevention scores. Blood samples (3.0 mL) were collected from each participant to test for HBV serum markers using a Wondfo One Step HBV rapid immunochromatographic assay (Catalog number W003) for the Hepatitis B surface antigen (HBsAg) profile. Data was analyzed using Stata/SE 15. Descriptive statistics were used for the socio-demographic, prevalence, and awareness variables. Chi-square and Fisher´s Exact Test were performed to determine the association between the variables.” was partially implemented. There remain the issue of the study design not attended to. The error of a “prospective” cross-sectional design is in error. This raises the question of What qualifies this study protocol to be considered a prospective study? Were data collected repeatedly throughout the period of the study for every participant?

**Response:** We sincerely apologised for this oversight. Please we have made the changes to the study design to reflect the suggestions by the reviewers. It now reads “*A cross-sectional study employing systematic random sampling was conducted among 183 consented pregnant women who went for antenatal care in nine health facilities in the Wa Municipality. A structured validated questionnaire was used to collect information about socio-demographic and obstetric characteristics, awareness of HBV transmission and its prevention. Blood samples (3.0 mls) were collected from each participant to test for HBV serum markers using a Wondfo One Step HBV rapid immunochromatographic assay (Catalog number W003) for the Hepatitis B surface antigen (HBsAg). We conducted descriptive statistics including the prevalence and used multivariable logistic regression to determine the risk of Hepatitis B among study participants*”

3) The flaws appearing in the result presented in the abstract was pointed out in the first round of the review process but it appeared that no action was taken to revise the flawed observations.

**Response:** We apologise for not accurately addressing this earlier on and that has been addressed now. It reads “*Generally, compared with younger pregnant women, older (>=25) pregnant women were >9 times less likely to test positive for both chronic (HBcAb) and (HBeAg) Hepatitis B infections. However, pregnant women in polygamous relationship were more likely to test positive for both (HBcAb) and (HBsAg and HBeAg) Hepatitis B infections compared with those in monogamous relationship. In a multivariable analysis, pregnant women in a polygamous relationships were about 5 times more likely to test positive for HBsAg (AOR=4.61, 95% CI: 2.06-9.89) and HBcAb (AOR=4.89, 95% CI:1.52-6.81) and HBeAg (AOR=4.62, 95% CI:1.21-6.39) compared with those in a monogamous relationship*.”

4) There is gross misapplications of 95%CI for p-values throughout the results presented. Kindly note the data in line 34-35: “…monogamy (95%CI [.01-.03], p<0.01)…” appear to have missed the sample statistic that should have been placed before the 95%CI.

**Response:** Thank you for spotting these important errors. We have made the corrections and added more details on both the univariable and multiple variable logistics regression analyses.

At the data analysis section, we added “*To evaluate associations with Hepatitis B serological markers, we used logistic regressions to obtain Odds Ratio (OR) along with 95% confidence intervals (CI). To identify the factors that remained independently associated with the Hepatitis B serological markers (our outcomes), we fitted a single multivariable logistics regression model that, at the onset, included all covariates and potential confounders for which a Wald test of their estimated coefficient yielded p<0.05 in the univariable logistics analyses. This resulted in estimation of Adjusted Odds Ratio (AOR) with 95% CI. The model goodness of fit was assessed based on low Akaike Information Criterion (AIC), and Hosmer-Lemeshow test.”*

At the result section, we added *“In our univariate analyses (****Table 4****), occupation, marital status, gravida, gestation, educational level, residence, history of blood transfusion, body piercing, tattooing and family history of Hepatitis B, residence are not significantly associated with a positive test for HBsAg, HBcAb, HBsAb, and HBeAb. However, age and marital type are significantly associated with a positive test for HBsAg, HBeAg, and HBcAb. For instance, pregnant women aged 25-34 (AOR=0.61, 95% CI:0.27-0.89) and ≥35 (AOR=0.59, 95% CI:0.39-0.68) years were less likely to test positive for HBeAg compared with pregnant women age between 15-24 years.*

*From* ***Table 5****, our multivariable logistic regression analyses found that pregnant women aged 25-34 (AOR=0.21, 95% CI:0.01-0.78) and ≥35 (AOR=0.24, 95% CI:0.05-0.62) years were less likely to test positive for HBeAg compared with pregnant women age between 15-24 years. This was similar with HBcAb were women age between 25-34 (AOR=0.42, 95% CI: 0.09-0.80) and ≥35 (AOR=0.31, 95% CI:0.06-0.45) were less likely to test positive compared with pregnant women age between 15-24 years.*

*On the contrary, pregnant women in the polygamous relationship were 5 times more likely to test positive for HBsAg (AOR=4.61, 95% CI: 2.06-9.89), HBcAb (AOR=4.89, 95% CI:1.52-6.81), and HBeAg (AOR=4.62, 95% CI: 1.21-6.39) compared to pregnant women in monogamous relationship.”*

5) The conclusion in lines 39 - 40. do not emerge from the data. What the data suggest is that “...a high HBsAg prevalence, is likely involved with the type of marital relationship and young age.” rather than influenced because the data analysis was more of comparing the likelihood of an outcome of risk. This well expressed in line 36 - 38. The logistic regression is most appropriate in establishing predictive value when comparing explanatory variables likely to predict an outcome than its current misuse for association. To establish and characterize the nature of association would be best to apply multiple regression.

**Response:** Thank you for this comment. We have corrected this error. We have also conducted both univariable and multivariable logistics regressions to identify the factors associated with the Hepatitis B virus serological biomarkers. The detail on analyses and results have been reported in the “data analysis” and “results” sections as shown in response# 4.

6) Kindly read through the manuscript for typographical errors and correct them.

**Response:** Thank you for the comment. We have read through the manuscript thoroughly and corrected all typographical errors as suggested.
